# Complete genome sequence of *Haliangium ochraceum* type strain (SMP-2^T^)

**DOI:** 10.4056/sigs.69.1277

**Published:** 2010-01-28

**Authors:** Natalia Ivanova, Chris Daum, Elke Lang, Birte Abt, Markus Kopitz, Elizabeth Saunders, Alla Lapidus, Susan Lucas, Tijana Glavina Del Rio, Matt Nolan, Hope Tice, Alex Copeland, Jan-Fang Cheng, Feng Chen, David Bruce, Lynne Goodwin, Sam Pitluck, Konstantinos Mavromatis, Amrita Pati, Natalia Mikhailova, Amy Chen, Krishna Palaniappan, Miriam Land, Loren Hauser, Yun-Juan Chang, Cynthia D. Jeffries, John C. Detter, Thomas Brettin, Manfred Rohde, Markus Göker, Jim Bristow, Victor Markowitz, Jonathan A. Eisen, Philip Hugenholtz, Nikos C. Kyrpides, Hans-Peter Klenk

**Affiliations:** 1 DOE Joint Genome Institute, Walnut Creek, California, USA; 2 DSMZ – German Collection of Microorganisms and Cell Cultures GmbH, Braunschweig, Germany; 3 Los Alamos National Laboratory, Bioscience Division, Los Alamos, New Mexico, USA; 4 Biological Data Management and Technology Center, Lawrence Berkeley National Laboratory, Berkeley, California, USA; 5 Oak Ridge National Laboratory, Oak Ridge, Tennessee, USA; 6 HZI – Helmholtz Centre for Infection Research, Braunschweig, Germany; 7 University of California Davis Genome Center, Davis, California, USA

**Keywords:** aerobic, gliding, myxobacteria, fruiting bodies, moderately halophilic, mesophile, Gram-negative, decomposition of bacterial and yeast cells, *Myxococcales*, GEBA

## Abstract

*Haliangium ochraceum* Fudou et al. 2002 is the type species of the genus *Haliangium* in the myxococcal family ‘*Haliangiaceae*’. Members of the genus *Haliangium* are the first halophilic myxobacterial taxa described. The cells of the species follow a multicellular lifestyle in highly organized biofilms, called swarms, they decompose bacterial and yeast cells as most myxobacteria do. The fruiting bodies contain particularly small coccoid myxospores. *H. ochraceum* encodes the first actin homologue identified in a bacterial genome. Here we describe the features of this organism, together with the complete genome sequence, and annotation. This is the first complete genome sequence of a member of the myxococcal suborder *Nannocystineae*, and the 9,446,314 bp long single replicon genome with its 6,898 protein-coding and 53 RNA genes is part of the *** G****enomic* *** E****ncyclopedia of* *** B****acteria and* *** A****rchaea * project.

## Introduction

Strain SMP-2^T^ (DSM 14365 = CIP 107738 = JCM 11303) is the type strain of the species *Haliangium ochraceum* and was first described in 2002 by Fudou et al. [1]. In 1998 strain SMP-2^T^ was described as swarming myxobacteria-like microorganism isolated from a dry seaweed sample (*Laminariales*)with optimum growth at NaCl concentrations of 2%. The attempt to isolate halophilic myxobacteria was initiated by the detection of myxobacterial phylotypes in marine sediments [2]. A second species of the genus *Haliangium*, *H. tepidum*, was described along with *H. ochraceum* [1].

Only two other genera of marine myxobacteria, each comprising one species, have been described to date: *Plesiocystis pacifica* and *Enhygromyxa salina* [3,4]. All marine myxobacteria are phylogenetically grouped within one of the three suborders within the order *Myxococcales*, the *Nannocystineae*. INSDC databases indicate (as of December 2009) that members of *Haliangium* are very rare in the environment, with the most closely related 16S rRNA gene sequences from uncultured bacteria being less than 94% similar to *H. ochraceum* SMP-2^T^.

## Classification and features

At the time of species description of the two *Haliangium* species, the most similar 16S rRNA gene sequence from cultivated strains originated from strain Pl vt1^T^. This strain was published with the name *Polyangium vitellinum* [5], hence the accession entry of its sequence (AJ233944) was also registered with this species name up to November 2009. However, Reichenbach perceived that these organisms meet perfectly Kofler’s description of “*Polyangium flavum*”, but do not conform to the description of the genus *Polyangium*. Thus Reichenbach revived Kofler’s “*Polyangium flavum*” in a new genus, *Kofleria*, and designated strain Pl vt1^T^ the type strain of the species *Kofleria flava* [6]. Subsequently, the species name was changed in the Genbank entry for AJ233944. The 16S rRNA gene sequences of the two *Haliangium* species were less than 94% similar to this nearest neighbor [1], and thus far no sequences of cultivated or uncultivated bacteria with higher similarities to SMP-2^T^ were deposited in GenBank.

In 2005, the family *Kofleriaceae* was created by Reichenbach, containing the single species *K. flava* [6], and the author mentioned in a note added during the edition of Bergey’s Manual that he regarded the two *Haliangium* species as members of the family *Kofleriaceae*. This family name has standing in nomenclature [7]. Albeit, *Haliangium ochraceum* is listed in the Taxonomic Outline of the Prokaryotes [8] as member of the family “*Haliangiaceae*”, that has no standing in nomenclature. From a phylogenetic point of view, the genera *Kofleria* (terrestrial) and *Haliangium* (marine) should be members of a single family.

Myxobacteria are distinct because of two exceptional features. The first is their high potential to produce secondary metabolites, most of them affecting prokaryotic or eukaryotic cells and hence awaiting exploitation for pharmaceutical applications or in plant protection. They encode genes for key enzymes in the biosynthesis of polyketide and peptide metabolites, polyketide synthases and nonribosomal peptide synthetases, respectively [9]. Their second distinctive characteristic is their morphogenesis, i.e. the formation of fruiting bodies and development of myxospores, that is based on cell-to-cell signaling among the single cells of the population in a swarm. The genetic background of the so called ‘social motility’ and morphogenesis is understood best for *Myxococcus xanthus* [10]. It is no surprise that these phenomena are regulated by sophisticated networks including two-component regulatory systems [11].

**Figure 1:**
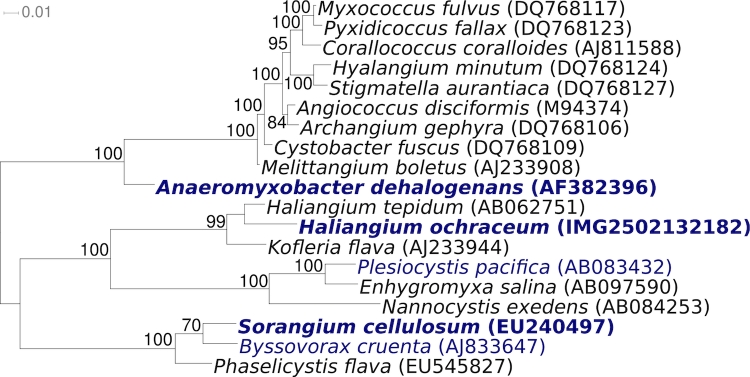
Phylogenetic tree highlighting the position of *H. ochraceum* SMP-2^T^ relative to the other type strains within the genus and the type strains of the other genera within the order Myxococcales. The tree was inferred from 1,463 aligned characters [12,13] of the 16S rRNA gene sequence under the maximum likelihood criterion [14] and rooted in accordance with the current taxonomy. The branches are scaled in terms of the expected number of substitutions per site. Numbers above branches are support values from 1,000 bootstrap replicates if larger than 60%. Lineages with type strain genome sequencing projects registered in GOLD [15] are shown in blue, published genomes in bold.

Figure 1 shows the phylogenetic neighborhood of *H. ochraceum* SMP-2^T^ in a 16S rRNA based tree. The sequences of the two 16S rRNA gene copies in the genome of do not differ from each other, and do not differ from the previously published 16S rRNA sequence of DSM 14365 (AB016470).

**Figure 2:**
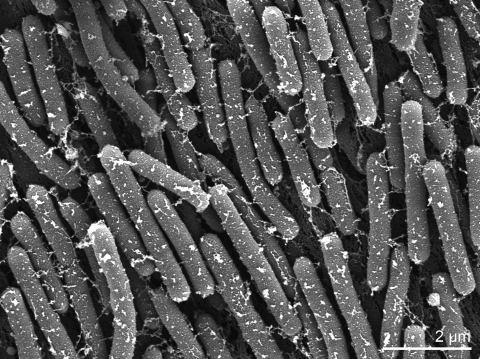
Scanning electron micrograph of *H. ochraceum* SMP-2^T^

Vegetative cells of *H. ochraceum* stain Gram-negative and form cylindrical rods with blunt ends (Table 1). They are embedded in an extracellular matrix and measure 0.5-0.6 by 3-8 µm (Figure 2). This cell form is characteristic for members of the suborder *Nannocystineae* [6]. The colonies exhibit spreading on solid surfaces such as agar as film-like layers and thus are called ‘swarms’. The extending motion is propelled by gliding. On aging culture plates, the cells do no more spread to explore new substrates (so called adventurous or A motility) but also gather on specific points of the swarms to form fruiting bodies (social or S motility) [10]. The fruiting bodies of strain SMP-2^T^ are light yellow to yellowish-brown, irregular, sessile knobs with a diameter of 50-200 µm and contain one or more oval-shaped sporangioles, each 20-60 µm in size [1,2]. The spherical to ovoid myxospores within the sporangioles measure 0.5-0.7 µm. Thus they resemble the myxospores of *Nannocystis* species in being very tiny [1]. The myxospores tolerate heat treatment at 55-60°C for 5 minutes and storage in a desiccated stage for at least 3 months [23].

**Table 1:** Classification and general features of *H. ochraceum* SMP-2^T^ according to the MIGS recommendations [16]

**MIGS ID**	**Property**	**Term**	**Evidence code**
	Current classification	Domain *Bacteria*	TAS [17]
		Phylum *Proteobacteria*	TAS [18]
		Class *Deltaproteobacteria*	TAS [19]
		Order *Myxococcales*	TAS [20,21]
		Suborder *Nannocystineae*	TAS [6,22]
		Family *‘Haliangiaceae’/Kofleriaceae*	TAS [6,8]
		Genus *Haliangium*	TAS [1]
		Species *Haliangium ocharaceum*	TAS [1]
		Type strain SMP-2	TAS [1]
	Gram stain	negative	TAS [1]
	Cell shape	rods	TAS [1]
	Motility	gliding	TAS [1]
	Sporulation	myxospores	TAS [1]
	Temperature range	mesophile, 20-40°C	TAS [1]
	Optimum temperature	30-34°C	TAS [1]
	Salinity	halophile, optimum 2% NaCl	TAS [1]
		tolerates up to 6% NaCl	TAS [2,23]
MIGS-22	Oxygen requirement	strictly aerobic	TAS [1]
	Carbon source	macromolecules such as proteins	TAS [1]
	Energy source	chemoorganotrophic	TAS [1]
MIGS-6	Habitat	marine	TAS [1,23]
MIGS-15	Biotic relationship	isolated from seaweed	TAS [2]
MIGS-14	Pathogenicity	non pathogenic	NAS
	Biosafety level	1	TAS [24]
	Isolation	dry sample of seaweed (*Laminariales*)	TAS [1]
MIGS-4	Geographic location	Miura Peninsula, Japan	TAS [2]
MIGS-5	Sample collection time	1997	TAS [2]
MIGS-4.1 MIGS-4.2	Latitude, Longitude	35.259, 139.629	NAS
MIGS-4.3	Depth	not reported	
MIGS-4.4	Altitude	sea-level	

Evidence codes - IDA: Inferred from Direct Assay (first time in publication); TAS: Traceable Author Statement (i.e., a direct report exists in the literature); NAS: Non-traceable Author Statement (i.e., not directly observed for the living, isolated sample, but based on a generally accepted property for the species, or anecdotal evidence). These evidence codes are from of the Gene Ontology project [25]. If the evidence code is IDA, then the property was directly observed for a live isolate by one of the authors or an expert mentioned in the acknowledgements.

The strain requires NaCl for growth with an optimum concentration of 2% and good growth in the range of 0.5-4% NaCl in agar or in liquid medium [1,2,23]. Fruiting body formation was observed at salt concentrations corresponding to 40-100% sea water concentration but not at lower salt concentrations [23]. Media supporting growth are CY medium, diluted 1:5, (DSMZ medium 67) or VY/2 medium (DSMZ medium 9) [26], each supplemented with seawater salts. No growth was obtained in tryptic soy broth with seawater salts [1]. Corresponding to the multicellular lifestyle, new agar or liquid cultures of strain SMP-2^T^ can only be successfully started with very high inoccula. The minimum cell load on a plate in order to induce a swarm is 105 [23]. The temperature range for growth is 20-40°C with an optimum at 30-34°C [1].

Cells of strain SMP-2^T^ are strictly aerobic with weak oxidase and catalase reactions. They do not grow in mineral media with carbohydrates or organic acids but are specialized decomposers of macromolecules such as starch, DNA, casein, chitin or gelatin. Cellulose, however is not cleaved. The cells are equipped to decompose cells of other bacteria or yeasts [1]. Correspondingly, enzymes such as lipase (C14), trypsin, chymotrypsin, valine or leucine arylamidases are active [1]. Whether or not *H. ochraceum* actively hunt for prey bacteria as shown for *M. xanthus* [27] has not been studied yet.

### Chemotaxonomy

The fatty acid profile of strain SMP-2^T^ reveals saturated straight chain C_16:0_ (38.3%) and branched chain iso-C_16:0_ (15.3%) acids as the major fatty acids. No hydroxylated fatty acids were detected, a feature shared with members of the genera *Nannocystis*, *Sorangium* [1], *Plesiocystis* [4] and *Enhygromyxa* [3]. While the two *Haliangium* species also contain anteiso-branched fatty acids as distinctive compounds [1], the specific feature of the two other marine genera *Plesiocystis* and *Enhygromyxa* is the presence of polyunsaturated C_20:4_ acids [3,4]. A novel pathway for the biosynthesis of iso-even fatty acids (by α-oxidation of iso-odd fatty acids) was detected for the myxobacterium *Stigmatella aurantiaca* [28]. In members of the genus *Nannocystis* and *Polyangium*, true steroids were detected, a very unusual trait among prokaryotes [6,29]. It would be interesting to study whether these pathways are also found in *H. ochraceum*.

MK-8 is the predominant menaquinone in SMP-2^T^ as it is in all terrestrial myxobacterial taxa studied [1,29]. It is noteworthy that the members of the other marine genera *Plesiocystis* and *Enhygromyxa* contain MK-8(H_2_) and MK-7, respectively [3,4]. The compositions of polyamines and the polar lipids of *Haliangium* strains have not been analyzed.

## Genome sequencing and annotation

### Genome project history

This organism was selected for sequencing on the basis of its phylogenetic position, and is part of the *** G****enomic* *** E****ncyclopedia of* *** B****acteria and* *** A****rchaea * project [30]. The genome project is deposited in the Genome OnLine Database [15] and the complete genome sequence is deposited in GenBank. Sequencing, finishing and annotation were performed by the DOE Joint Genome Institute (JGI). A summary of the project information is shown in Table 2.

**Table 2:** Genome sequencing project information

**MIGS ID**	**Property**	**Term**
MIGS-31	Finishing quality	Finished
MIGS-28	Libraries used	Two Sanger genomic libraries – 6 kb pMCL200 and fosmid pcc1Fos and one 454 pyrosequencing standard library
MIGS-29	Sequencing platforms	ABI3730, 454 GS FLX
MIGS-31.2	Sequencing coverage	7.8× Sanger; 16.5× pyrosequence
MIGS-30	Assemblers	Newbler version 1.1.02.15, phrap
MIGS-32	Gene calling method	Prodigal, GenePRIMP
	INSDC ID	CP001804
	Genbank Date of Release	October 28, 2009
	GOLD ID	Gc01135
	NCBI project ID	41425
	Database: IMG-GEBA	2502082105
MIGS-13	Source material identifier	DSM 14365
	Project relevance	Tree of Life, GEBA

### Growth conditions and DNA isolation

*H. ochraceum* SMP-2^T^, DSM 14365, was grown in CY medium with seawater salts (in grams per liter: casitone 3.0, yeast extract 1.0, NaCl 21.1, KCl 0.6, CaCl_2_ × 2 H_2_O 1.2, MgCl_2_ × 6 H_2_O 3.6, NaHCO_3_ 0.09, MgSO_4_ × 7H_2_O 2.6, agar 15 g) [26] at 28°C. DNA was isolated from 0.5-1 g of cell paste using Qiagen Genomic 500 DNA Kit (Qiagen, Hilden, Germany), with a modified protocol for cell lysis (st/LALMP), as described in Wu *et al*. [30].

### Genome sequencing and assembly

The genome was sequenced using a combination of Sanger and 454 sequencing platforms. All general aspects of library construction and sequencing performed at the JGI can be found at the JGI website (http://www.jgi.doe.gov/). 454 Pyrosequencing reads were assembled using the Newbler assembler version 1.1.02.15 (Roche). Large Newbler contigs were broken into 10,273 overlapping fragments of 1,000 bp and entered into the final assembly as pseudo-reads. The sequences were assigned quality scores based on Newbler consensus q-scores with modifications to account for overlap redundancy and to adjust inflated q-scores. A hybrid 454/Sanger assembly was made using the parallel phrap assembler (High Performance Software, LLC). Possible mis-assemblies were corrected with Dupfinisher or transposon bombing of bridging clones [31]. Gaps between contigs were closed by editing in Consed, custom primer walk or PCR amplification. A total of 2,013 Sanger finishing reads were produced to close gaps, to resolve repetitive regions, and to raise the quality of the finished sequence. The error rate of the completed genome sequence is less than 1 in 100,000. Together all sequence types provided 24.3× coverage of the genome. The final assembly contains 90,757 Sanger and 689,516 pyrosequencing reads.

### Genome annotation

Genes were identified using Prodigal [32] as part of the Oak Ridge National Laboratory genome annotation pipeline, followed by a round of manual curation using the JGI GenePRIMP pipeline (http://geneprimp.jgi-psf.org/) [33]. The predicted CDSs were translated and used to search the National Center for Biotechnology Information (NCBI) nonredundant database, UniProt, TIGRFam, Pfam, PRIAM, KEGG, COG, and InterPro databases. Additional gene prediction analysis and functional annotation was performed within the Integrated Microbial Genomes - Expert Review (http://img.jgi.doe.gov/er) platform [34].

### Genome properties

**Figure 3:**
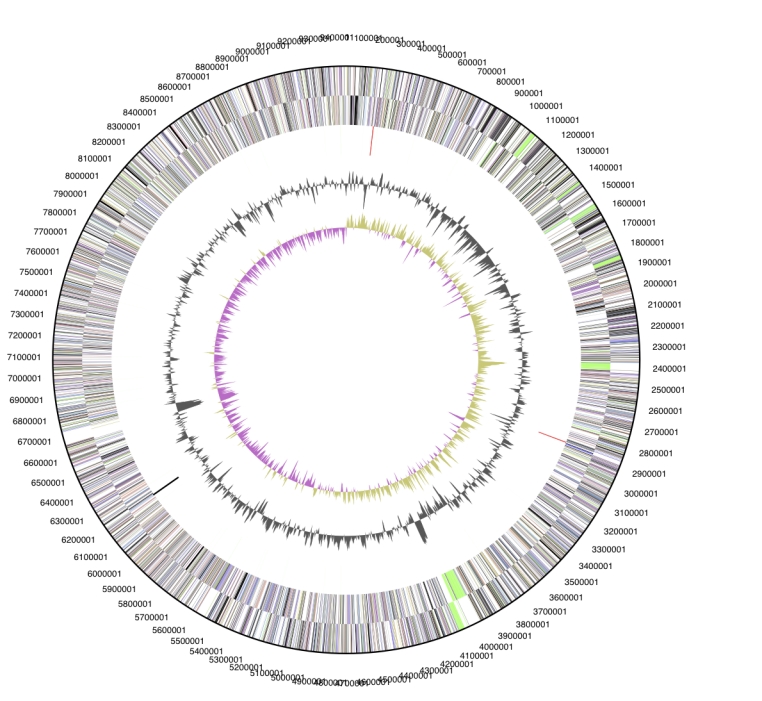
Graphical circular map of the genome. From outside to the center: Genes on forward strand (color by COG categories), Genes on reverse strand (color by COG categories), RNA genes (tRNAs green, rRNAs red, other RNAs black), GC content, GC skew.

The genome is 9,446,314 bp long and comprises one main circular chromosome with a 69.5% GC content (Table 3 and Figure 3). Of the 6,951 genes predicted, 6,898 were protein coding genes, and 53 RNAs. Fifty-three pseudogenes were also identified. The majority of the protein-coding genes (62.1%) were assigned with a putative function while the remaining ones were annotated as hypothetical proteins. The percentage of genes which were not assigned to COGs is relatively high, 42%, a proportion similar to that in the genome of *Sorangium cellulosum* So ce56 [11]. This fact suggests that the genome harbors many yet unknown genes. The distribution of genes into COGs functional categories is presented in Table 4.

**Table 3:** Genome Statistics

**Attribute**	Value	% of Total
Genome size (bp)	9,446,314	100.00%
DNA coding region (bp)	8,424,350	89.18%
DNA G+C content (bp)	6,563,619	69.48%
Number of replicons	1	
Extrachromosomal elements	0	
Total genes	6,951	100.00%
RNA genes	53	0.76%
rRNA operons	2	
Protein-coding genes	6,898	99.24%
Pseudo genes	53	0.76%
Genes with function prediction	4,318	62.12%
Genes in paralog clusters	1,329	19.12%
Genes assigned to COGs	4,036	58.06%
Genes assigned Pfam domains	4,167	59.95%
Genes with signal peptides	1,786	25.69%
Genes with transmembrane helices	1,371	19.72%
CRISPR repeats	3	

**Table 4:** Number of genes associated with the general COG functional categories

**Code**	**Value**	**%age**	**Description**
J	182	2.6	Translation, ribosomal structure and biogenesis
A	2	0.0	RNA processing and modification
K	488	7.1	Transcription
L	439	6.4	Replication, recombination and repair
B	3	0.0	Chromatin structure and dynamics
D	54	0.8	Cell cycle control, mitosis and meiosis
Y	0	0.0	Nuclear structure
V	83	1.2	Defense mechanisms
T	549	8.0	Signal transduction mechanisms
M	250	3.6	Cell wall/membrane biogenesis
N	55	0.8	Cell motility
Z	16	0.2	Cytoskeleton
W	0	0.0	Extracellular structures
U	89	1.3	Intracellular trafficking and secretion
O	194	2.8	Posttranslational modification, protein turnover, chaperones
C	265	3.8	Energy production and conversion
G	186	2.7	Carbohydrate transport and metabolism
E	308	4.5	Amino acid transport and metabolism
F	78	1.1	Nucleotide transport and metabolism
H	183	2.7	Coenzyme transport and metabolism
I	204	3.0	Lipid transport and metabolism
P	163	2.4	Inorganic ion transport and metabolism
Q	174	2.5	Secondary metabolites biosynthesis, transport and catabolism
R	754	10.9	General function prediction only
S	332	4.8	Function unknown
-	2915	42.3	Not in COGs

Starting from one of the conspicuous features of the myxobacteria, the diversity of secondary metabolism, the number of known genes putatively assigned to the COG category “Secondary metabolites biosynthesis, transport and catabolism” is not exceptionally high: 174 genes in comparison to, for example, 136 genes in *Pseudomonas putida* F1. The number of COG genes involved in “Replication, recombination and repair”, however, are remarkably increased: in *H. ochraceum*: 439 genes were assigned to this category, in *S. cellulosum* there are 541, whereas *P. putida* only contains 157 genes assigned to this category.

## Insights from genome sequence

The genomes of two other myxobacteria, *M. xanthus* DK1622 and *S. cellulosum* strain So ce56, were analyzed in depth [11,35-37] and may serve as a roadmap to explore the genome of strain SMP-2^T^.

Sixteen genes of strain SMP-2^T^ were putatively assigned to the COG category ‘Cytoskeleton’. Recognizing that almost all other bacteria do not harbor any genes assigned to this category it is worth mentioning that all myxobacterial genomes studied so far include several copies in this category. Fifteen of the cytoskeleton genes of SMP-2^T^ belong to COG 5184 ’Alpha-tubulin suppressor and related RCC1 domain-containing proteins‘. Strain SMP-2^T^ and *P. pacifica*, another rare marine myxobacterium, together with *Salinispora arenicola* are the prokaryotes with the highest degree of similarity of these genes, 15, 12, 14 and 15, respectively. Whereas RCC1 was known as a eukaryotic cell cycle regulator, RCC1-like repeats were recently also detected in several prokaryotic genomes [38]. Future studies will have to elucidate whether the SMP-2^T^ sequences, automatically assigned to a RCC1 domain, are related to these repeats in particular. As the genes most similar to the *H. ochraceum* RCC1-like proteins, as determined by protein BLAST with the NCBI database, derive exclusively from other myxobacteria such as *P. pacifica* or *Stigmatella aurantiaca*, it seems plausible that they build a myxobacterial branch within the RCC1 superfamily.

The most striking finding in the *H. ochraceum* genome was a sequence coding for a protein of the actin family (COG 5277) within the Cytoskeleton category [30]. Only eight years ago, it became obvious that bacterial cells contain a cytoskeleton at least as active as in eukaryotic cells. The bacterial functional and structural homologues to the eukaryotic actin compound are the proteins MreB and ParM [39]. However, the prokaryotic and eukaryotic genes coding for these proteins, or their amino acid sequences, are not related on the sequence level. In contrast, the sequence detected in *H. ochraceum* shows a striking sequence similarity to actin and is the very first report of an actin homolog in a bacterial genome. The protein was called BARP, bacterial actin-related protein. The genomic context of barP, its sequence, the putative structure of the protein and evidence that the gene is expressed were recently described by Wu *et al*. [30]. Interestingly, several hits for proteins of the actin family are given for *Archaea* by IMG.

Myxobacteria became known for their potential to synthesize a vast array of secondary metabolites. Polyketide synthases (PKS) and nonribosomal peptide synthetases play the key role in the building pathways [37]. PKS multidomain complexes are listed in COG 3221 in the category ‘Secondary metabolites’. The sum of automatic assignments to this category is not extraordinarily increased for *H. ochraceum* in comparison to other bacteria (174 hits as compared to, e.g., 136 in *P. putida* strain F1), and the search for the gene product ‘polyketide synthase’ does not find any gene for *H. ochraceum*. However, the genome of *H. ochraceum*, like the other myxobacteria studied, contains a high number of stretches assigned to COG 3321. The number of hits in COG is less than 10 in bacteria except for the myxobacteria, *Burkholderia mallei, B. pseudomallei*, *Mycobacterium* spp. and members of the *Streptomyces*. The annotations in COG 3321 for *H. ochraceum* identify the homologues as known domains of PKS (for example acyltransferases or ketoreductases) or of a distinct PKS synthesizing the aglycone precursor of erythromycin B. A search for PKS in different myxobacteria using PCR unfortunately did not include *H. ochraceum* but it included a strain representing the second species in the genus, *H. tepidum* [40]. The authors found the highest percentage of yet undescribed PKS sequences (50% of all newly detected PKS sequences) in the marine myxobacteria (as compared to terrestrial myxobacteria). In *H. tepidum*, all 10 PKS sequences represented novel PKS genes (threshold 70% identity to known sequences). These findings suggest that an in-depth search for novel genes coding for isoprenoid metabolites in *H. ochraceum* has a very good prospect of success.

Other promising fields of gene mining in *H. ochraceum*, as a representative of the marine myxobacteria, most likely are the genes of energy metabolism and the genes coding for the coordinated movement of cells during fruiting body and myxospore formation. This morphogenesis is conducted by cell-to-cell cross-talk, signal transduction and induction of ‘social motility’ [10,41].
